# A Prognostic Autophagy-Related Gene Pair Signature and Small-Molecule Drugs for Hepatocellular Carcinoma

**DOI:** 10.3389/fgene.2021.689801

**Published:** 2021-08-23

**Authors:** ZeBing Song, GuoPei Zhang, Yang Yu, ShaoQiang Li

**Affiliations:** Department of Liver Surgery, The First Affiliated Hospital of Sun Yat-sen University, Guangzhou, China

**Keywords:** autophagy-related gene pair signature, small-molecule drugs, hepatocellular carcinoma, bioinformatic analysis, autophagy

## Abstract

Dysregulation of autophagy-related genes (ARGs) is related to the prognosis of cancers. However, the aberrant expression of ARGs signature in the prognosis of hepatocellular carcinoma (HCC) remain unclear. Using The Cancer Genome Atlas and the International Cancer Genome Consortium database, 188 common autophagy-related gene pairs (ARGPs) were identified. Through univariate, least absolute shrinkage and selection operator analysis, and multivariate Cox regression analysis, a prognostic signature of the training set was constructed on the basis of 6 ARGPs. Further analysis revealed that the ARGP based signature performed more accurately in overall survival (OS) prediction compared to other published gene signatures. In addition, a high risk of HCC was closely related to CTLA4 upregulation, LC3 downregulation, low-response to axitinib, rapamycin, temsirolimus, docetaxel, metformin, and high-response to bleomycin. Univariate Cox and multivariate Cox analysis revealed that the risk score was an independent prognostic factor for HCC. These results were internally validated in the test and TCGA sets and externally validated in the ICGC set. A nomogram, consisting of the risk score and the TNM stage, performed well when compared to an ideal nomogram. In conclusion, a 6-ARGP-based prognostic signature was identified and validated as an effective predictor of OS of patients with HCC. Furthermore, we recognized six small-molecule drugs, which may be potentially effective in treating HCC.

## Introduction

Hepatocellular carcinoma (HCC) is one of the most common liver malignancies worldwide, with increasing rates of morbidity and mortality annually (Bray et al., 2018). The prognosis of patients with HCC is poor owing to its high recurrence rate. Hence, effective biomarkers are needed to help improve the prognosis of patients with HCC.

Autophagy, also called “programmed cell death type II,” plays an important role in *tumorigenesis*, metastasis and drug resistance (Huang et al., 2018). Liu K. et al. (2018) reported that autophagy is required for benign hepatic tumors to progress into malignant HCC. Another study revealed that the activation of autophagy can promote metastasis through the upregulation of MCT1 via activating Wnt/β-catenin signaling in HCC cells (Fan et al., 2018). Sorafenib is an effective molecular-targeted drug used to treat advanced-stage HCC by inducing autophagy, thus prolonging the survival of patients with HCC (Raza and Sood, 2014; Finn et al., 2018). While only approximately 30% of patients with advanced HCC respond well to sorafenib, resistance to sorafenib remains an open question, which may result from pro-survival pathways of autophagy induced by sorafenib (Sun T. et al., 2017; Jiang et al., 2018). These findings indicate that autophagy plays an important role in the prognosis of patients with HCC. Hence, autophagy-related genes (ARGs) can be effective biomarkers to diagnose, and guide treatments for HCC. However, some effective biomarkers to predict the prognosis of HCC have not been established thus far.

To construct an autophagy-related prognostic signature for HCC, The Cancer Genome Atlas (TCGA) and the International Cancer Genome Consortium (ICGC) data sets were used as the data source in this study. A 6-autophagy-related gene pair (ARGP) prognostic signature was identified and validated for its predictability of overall survival (OS) among patients with HCC in comparison with 3 others previously reported prognostic gene signatures. Furthermore, the effectiveness of small-molecule drugs for HCC was assessed, and 6 types of drugs were identified and validated in this study.

## Materials and Methods

### Data Source

**FIGURE 1:**
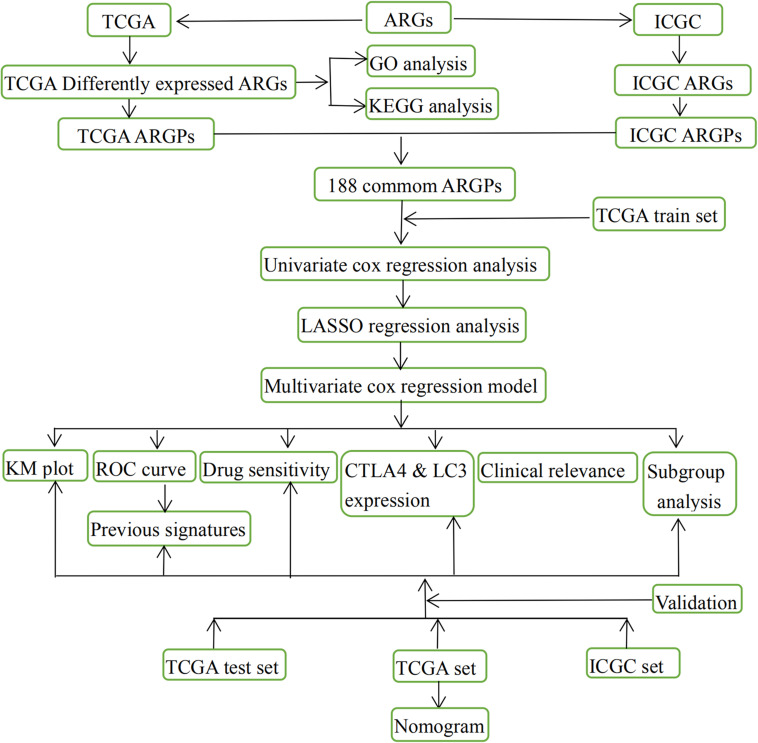
Entire workflow of the present study.

Transcription profiling RNA data, along with the HCC clinical data were downloaded from TCGA^1^ (Liu J. et al., 2018), and were used to identify differentially expressed ARGs. Finally, 370 patients with complete survival data were identified from TCGA and randomized into a training set (*n* = 185) and test set (*n* = 185); These two sets were used to develop and internally validate the HCC prognostic signature. The demographic characteristics of the training set, test set, and TCGA set are summarized in Table 1. To externally validate the prognostic value of the prognostic signature, the gene expression data, and the clinical data on patients with HCC from the ICGC database (ICGC-LIRI-JP)^2^ were downloaded as well. The databases selection and data procession flow chart of this study were shown in Figure 1.

**TABLE 1:** Clinicopathological parameters of hepatocellular carcinoma patients in training set, test set and TCGA data set.

Covariates	Type	Total	Train	Test	*P*-value
Age	≤65	232 (62.7%)	113 (61.08%)	119 (64.32%)	0.5909
	>65	138 (37.3%)	72 (38.92%)	66 (35.68%)	
	Unknow	0 (0%)	0 (0%)	0 (0%)	
Gender	Female	121 (32.7%)	68 (36.76%)	53 (28.65%)	0.1208
	Male	249 (67.3%)	117 (63.24%)	132 (71.35%)	
Grade	G1-2	232 (62.7%)	122 (65.95%)	110 (59.46%)	0.3227
	G3-4	133 (35.95%)	62 (33.51%)	71 (38.38%)	
	Unknow	5 (1.35%)	1 (0.54%)	4 (2.16%)	
Stage	I-II	256 (69.19%)	127 (68.65%)	129 (69.73%)	0.7612
	III-IV	90 (24.32%)	47 (25.41%)	43 (23.24%)	
	Unknow	24 (6.49%)	11 (5.95%)	13 (7.03%)	
T	T1-2	274 (74.05%)	137 (74.05%)	137 (74.05%)	0.9534
	T3-4	94 (25.41%)	46 (24.86%)	48 (25.95%)	
	Unknow	2 (0.54%)	2 (1.08%)	0 (0%)	
M	M0	266 (71.89%)	134 (72.43%)	132 (71.35%)	1
	M1	4 (1.08%)	2 (1.08%)	2 (1.08%)	
	Unknow	100 (27.03%)	49 (26.49%)	51 (27.57%)	
N	N0	252 (68.11%)	126 (68.11%)	126 (68.11%)	0.1387
	N1	4 (1.08%)	4 (2.16%)	0 (0%)	
	Unknow	114 (30.81%)	55 (29.73%)	59 (31.89%)	

### Identification of Differential Expression of ARGs Between HCC and Non-tumor Samples of TCGA

An ARG set was downloaded from the Human Autophagy Database^3^. We then extracted the expression profile of these ARGs on the basis of the gene set and TCGA transcription profiling data. Differentially expressed ARGs were identified using the limma package. Differential expression of ARGs with a log2 fold change (|log2FC|) > 1 and a false discovery rate < 0.05 were considered significant and were included in the subsequent analysis. The Gene Ontology (GO) and Kyoto Encyclopedia of Genes and Genomes (KEGG) functional analysis were performed to explore the potential molecular function of these differentially expressed ARGs (Bandyopadhyay and Mallick, 2014).

### Construction of a Prognostic Signature Based on the Training Set

In this study, OS was considered the primary endpoint. Pairwise comparison was performed between the expression profiles of differentially expressed ARGs in each HCC sample to obtain a score for each ARGP by using the R software. According to the proposed algorithm (Heinäniemi et al., 2013), if the expression level of the first ARG was higher than that of the second ARG in an ARGP, the score of this ARGP was 1; otherwise, the score was 0. If the score of an ARGP was 0 or 1 in > 80% of the samples of the training or the test sets, the ARGP was discarded, and the rest of the ARGPs were involved in subsequent analysis. Univariate Cox regression analysis was performed for the training set to identify OS-related ARGPs (FDR < 0.05), and the Least absolute shrinkage and selection operator (LASSO) analysis was performed to avoid overfitting of the prognostic signature. The most stable ARGP prognostic signature was constructed through multivariate Cox regression analysis (FDR < 0.05). In this study, patients were categorized into high- and low-risk groups in accordance with the median risk score. The risk score of the prognostic signature was calculated by multiplying the expression level with Cox regression coefficients of the ARGPs. The formula was as followed: risk score = Σ Cox regression coefficient of ARGPi ^∗^ expression value of gene ARGPi.

### Evaluation and Validation of the Prognostic Signature

The prognostic signature was evaluated by utilizing the training set and validated using the test set, TCGA set, and ICGC set. Following the median risk score of the train set, the patients were classified into high- and low-risk groups. The Kaplan-Meier (KM) method was applied to compare the OS between the high- and low-risk groups. The receiver operating characteristic (ROC) curve was plotted and the area under the curve (AUC) was calculated to ensure that the prognostic signature prediction efficacy can be estimated. Both univariate and multivariate Cox analysis were conducted with the clinicopathologic features and risk score to explore the HCC prognostic factors. Furthermore, we compared the AUC of the prognostic signature with that of 3 published gene prognostic signatures in the TCGA set. Subgroup analysis were performed to expand the application scope of the ARGP signature.

### Prediction of Potential Small-Molecule Drugs

The drug response toward axitinib, rapamycin, temsirolimus, docetaxel, metformin, and bleomycin in each patient with HCC in the training set, test set, TCGA set, and ICGC set was calculated on the basis of the Genomics of Drug Sensitivity in Cancer (GDSC)^4^ by using the prophetic R package^5^. The half maximal inhibitory concentration (IC50) value for patients with HCC was used to evaluate the effectiveness of these drugs, and *P* < 0.05 was set as the cutoff value.

### Establishment and Evaluation of a Nomogram for Predicting the Survival of Patients With HCC

We included all independent clinicalpathological prognostic factors selected from multivariate Cox regression analysis to construct a nomogram that can assess an OS probability of 1, 3, and 5 years for patients with HCC. The prediction probability of the nomogram was compared with the observed actual probability form the calibration curve to verify its accuracy. Overlaps with the reference line indicate that the model is accurate.

### Statistical Analysis

Statistical analysis was performed using the R software (version 3.6.3)^6^ and Perl software (version 5.30)^7^. Cluster heatmaps and volcano maps were generated using gplots and heatmap packages. Univariate and multivariable Cox proportional hazards regression analysis were performed using the survival R software package. The KM analysis was performed using the survival R package and assessed using the log-rank test (Aalen, 1988). The survival ROC R package was used to calculate the AUC of the survival ROC curve.

## Results

### Identification of Differential Expression of ARGs and Assessment of the Potential Molecular Function of ARGs

**FIGURE 2:**
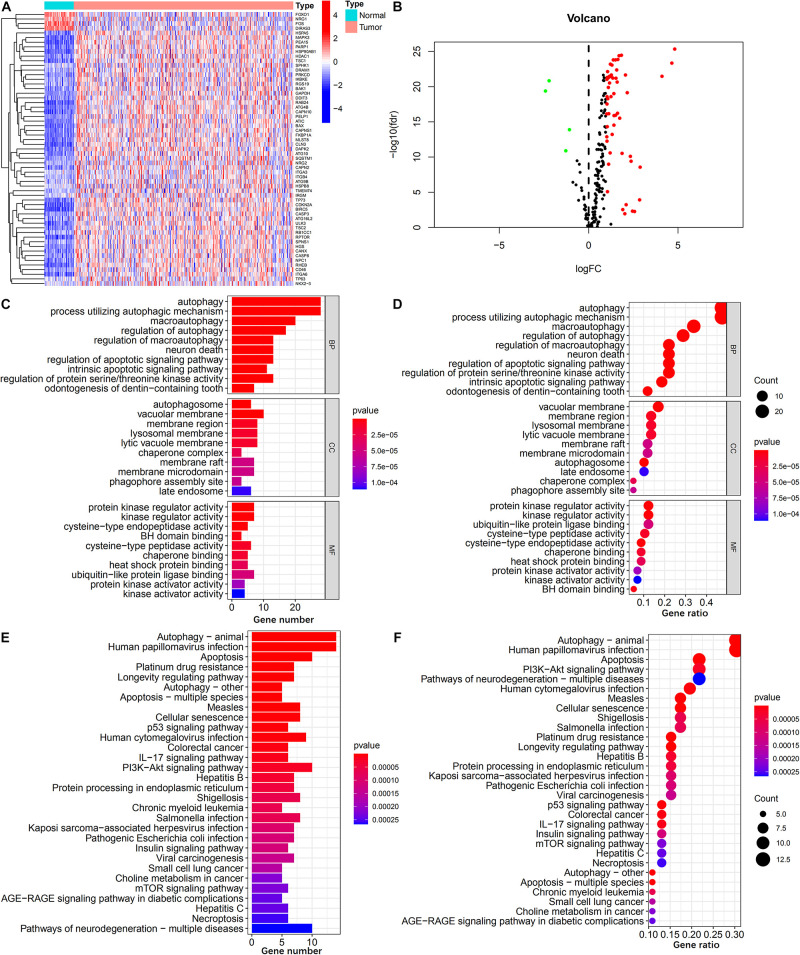
Identification of differentially expressed ARGs. **(A,B)** Heatmap and volcano plot illustrates the expression of 59 differentially expressed ARGs between HCC tumor and non-tumor specimens. **(C,D)** Barplot and bubble plot of GO analysis shows the top 10 biological functions of the differentially expressed ARGs in the biological processes, cellular components, and molecular functions. **(E,F)** Barplot and bubble plot of KEGG analysis shows the top 30 signaling pathways of the differentially expressed ARGs participated in.

As shown in Figures 2A,B 59 ARGs were identified, including 4 down-regulated and 55 up-regulated genes. GO analysis of these ARGs revealed that “autophagy,” “vacuolar membrane,” and “protein kinase regulator activity” were the most frequent biological terms for biological processes, cellular components, and molecular functions, respectively (Figures 2C,D; Wang et al., 2021). KEGG analysis revealed that the primary pathways of these ARGs were “autophagy-animal,” “IL-17 signaling pathway,” “PI3K-Akt signaling pathway,” and “mTOR signaling pathway,” which were primarily correlated with autophagy, immune process, and carcinogenicity (Figures 2E,F).

### Establishment of an ARGP Signature in the Training Set

**FIGURE 3:**
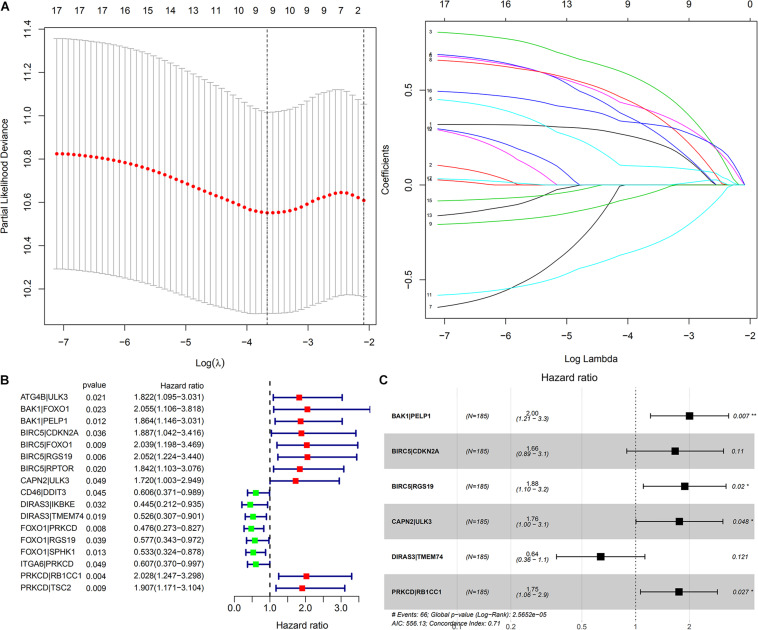
Construction of a prognostic signature based on ARGP. **(A)** The Cross-Validation fit curve was calculated by lasso regression analysis. **(B)** The Forest plot represents the 17 OS-related ARGPs identified by Univariate Cox regression analysis. **(C)** The 6 ARGPs to construct the prognostic signature selected by Multivariate Cox regression analysis. *P* < 0.05 sets as the cutoff value.

In total, 188 common ARGPs from among the TCGA and ICGC expression profile data were extracted. 17 ARGPs were found to be related to OS among patients with HCC, as revealed through univariate Cox regression analysis (Figure 3B). Thereafter, 9 ARGPs were found to be capable to construct a prognostic signature through LASSO analysis (Figure 3A). Finally, a 6-ARGP prognostic signature was constructed through multivariate Cox regression analysis, which included BAK1| PELP1, BIRC5| CDKN2A, BIRC5| RGS19, CAPN2| ULK3, DIRAS3| TMEM74, and PRKCD| RB1CC1 (Figure 3C). The risk score of our prognostic signature was as follows: risk score = (the expression level of BAK1| PELP1 ^∗^ 0.6951) + (the expression level of BIRC5| CDKN2A ^∗^ 0.5093) + (the expression level of BIRC5| RGS19 ^∗^ 0.6322) + (the expression level of CAPN2| ULK3 ^∗^ 0.5645) + (the expression level of DIRAS3| TMEM74 ^∗^ -0.4505) + (the expression level of PRKCD| RB1CC1 ^∗^ 0.5581).

**FIGURE 4:**
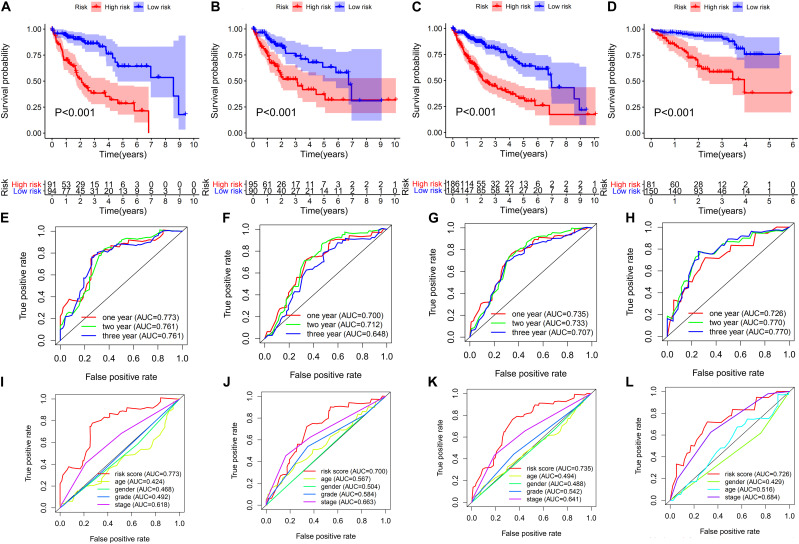
Evaluation and validation of the risk score of the 6-ARGP signature. Survival analysis and ROC analysis based on risk score in the training set **(A,E,I)**, test set **(B,F,J)**, TCGA set **(C,G,K)**, and ICGC set **(D,H,L)**, respectively.

The AUC of the 1-, 2-, and 3-year OS were 0.773, 0.761, and 0.761, respectively (Figure 4E). As shown in Figure 4A, the OS of the high-risk group was poorer than that of the low-risk group.

### Validation of the Predictive Values of the ARGP Prognostic Signature

The predictive values of the ARGP prognostic signature were validated in the test set, TCGA set, and ICGC set, respectively. To improve the accuracy of validation, patients in the aforementioned 3 data sets were randomized into high- and low-risk groups using the same cutoff of the training set, i.e., the median risk score of the training set. As shown in Figures 4B–D, the OS of the high-risk group of the aforementioned 3 data sets had poorer OS than the low-risk group. The AUC of test set, TCGA set and ICGC set was 0.7, 0.735, and 0.726 after year 1; 0.712, 0.733, and 0.77 after year 2; and 0.648, 0.707, and 0.77 after year 3, respectively (Figures 4F–H). In addition, the AUC of the ARGP signature was higher than age, gender, TNM stage and tumor grade in the train, test, TCGA and ICGC set (Figures 4I–L). The survival status of patients, the rank of the risk score, and the heatmap of expression profiles of the 6 ARGPs in the low- and high-risk groups are indicated in Supplementary Figure 1.

**FIGURE 5:**
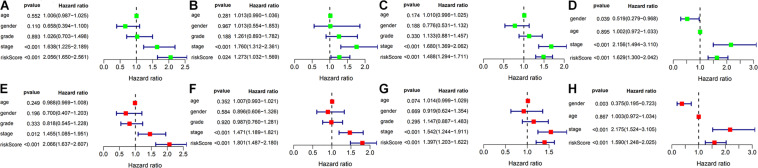
Univariate Cox and Multivariate Cox regression analysis of risk score and clinicopathologic factors in different cohorts. **(A–D)** Forest plot represents the results of univariate Cox regression analysis in the training set, test set, TCGA set and ICGC set, respectively. **(E–H)** Forest plot represents the results of multivariate Cox regression analysis in the training set, test set, TCGA set and ICGC set, respectively.

As shown in Figure 5, the univariate and multivariate Cox regression analysis indicate that the risk score could potentially be an independent prognostic factor after adjustment by age, gender, tumor grade, and TNM stage in the training (HR: 2.066, 95% CI: 1.637–2.607, *P* < 0.001), test (HR: 1.801, 95% CI: 1.487–2.18, *P* < 0.001), TCGA (HR: 1.397, 95% CI: 1.203–1.622, *P* < 0.001) and ICGC sets (HR: 1.59, 95% CI: 1.248–2.025, *P* < 0.001).

Subgroup analysis based on age (>65 and ≤ 65 years), gender (male and female), tumor grade (G1-2 and G3-4), TNM stage (I-II and III-IV), tumor stage (T1-2 and T3-4), lymph node metastasis status (N0), and *distant metastasis* status (M0) in the training, test, TCGA, and ICGC sets were performed to further validate the predictive values of the ARGP signature. As shown in Supplementary Figures 2, 3, all subgroup analysis in the training, TCGA, and ICGC sets performed well in OS prediction. In the test set, subgroup analysis performed well in OS prediction, except for patients in subgroups of female, G1-2, Stage III-IV, and T3-4.

As shown in Supplementary Figure 4, risk score of both the test and TCGA sets was associated with the pathological stage (I-II and III-IV), and tumor grade (G1-2 and G3-4), and the risk-score of training set was also correlated with the tumor grade (G1-2 and G3-4).

### Analysis of Cytotoxic T-Lymphocyte-Associated Protein 4 (CTLA4) and LC3 Expression Levels Between the High-Risk and Low-Risk Groups

**FIGURE 6:**
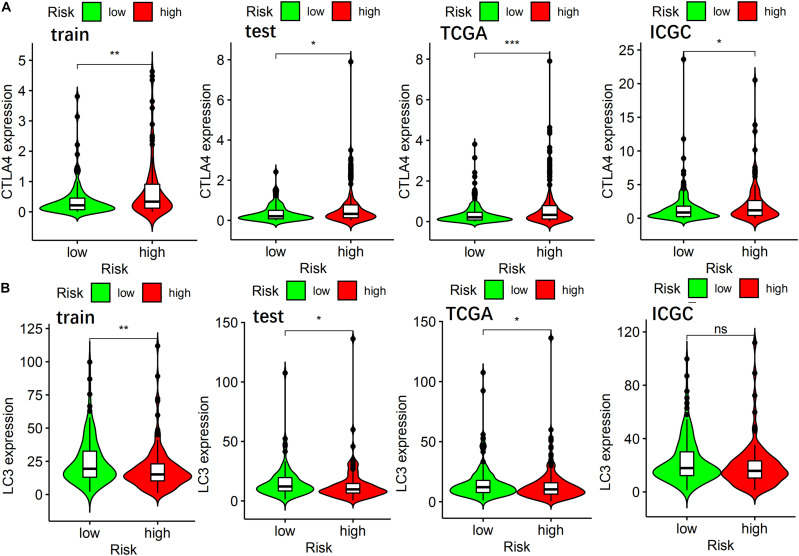
The relationship of ARGP risk group with CTLA4, LC3 expression in different data sets. **(A)** The differential expression of CTLA4 in the training set, test set, TCGA set, and ICGC set, respectively. **(B)** The differential expression of LC3 in the training set, test set, TCGA set, and ICGC set, respectively.

To further explore the role of autophagy and immune processes in the OS of patients with HCC, analysis of CTLA4 and LC3 expression levels between high-risk and a low-risk group of the ARGP prognostic signature was performed. As shown in Figure 6, the expression level of CTLA4 in the low-risk group of the training, test, TCGA, and ICGC sets was lower than that of the high-risk group, while the expression level of LC3 was higher in the low-risk group in the training, test, and TCGA sets.

### Drug Sensitivity Analysis Between High and Low-Risk Groups

As shown in Table 2, the IC50 value of axitinib, rapamycin, temsirolimus, docetaxel, and metformin in the low-risk group was lower than that in the high-risk group, indicating that these 5 small molecule drugs were more effective for patients in the low-risk group. However, the IC50 value of bleomycin was greater in the low-risk group than in the high-risk group, indicating that bleomycin was more effective for patients in the high-risk group.

**TABLE 2:** The relationship of ARGP risk group with small molecule drug therapy response in different data sets.

Drugs (IC50)	Group	Train	Test	TCGA	ICGC	*P*-value
Axitinib	High risk	3.95(−4to7.5)	3.2(−2.8to6.3)	3.75(−4.1to7.7)	3.8(−2.8to6.6)	<0.05
	Low risk	3(−3.6to7.6)	2.7(−2.6to6)	3(−3.8to7.6)	2.9(−4.9to6.5)	
Rapamycin	High risk	−0.15(−10to5)	0.04(−8to3.5)	−0.03(−9to4.3)	0.1(−2.5to2.4)	<0.05
	Low risk	−0.8(−4.8to4)	−1(−6.8to3.6)	−0.8(−7.5to4)	−0.5(−4.9to2.7)	
Temsirolimus	High risk	−0.1(−8to9.6)	−0.1(−8.9to9.1)	−0.1(−9to9.6)	0.3(−7to9.5)	<0.05
	Low risk	−2.3(−9to8.7)	−2.4(−9to9.3)	−2.3(−9to9.3)	−2.2(−9.6to7.8)	
Docetaxel	High risk	−5(−8.3to0)	−4.9(−8.7to0)	−5(−9to0)	−4.9(−6.3to0)	<0.05
	Low risk	−5.7(−9.4to1)	−5.7(−8.7to0)	−5.7(−9.4to1)	−5.8(−9.8to0)	
Metformin	High risk	11.5(7to14)	11(6.5to15)	11.5(6.5to15)	11.3(9to13.5)	<0.05
	Low risk	10(7.5to13.5)	10(6.5to12.5)	10(6.5to13.5)	10.2(7.5to13)	
Bleomycin	High risk	0.3(−11to9)	0.5(−10to7.5)	0.3(−11to9)	0.1(−9.6to5.4)	<0.05
	Low risk	4.3(−7to10)	4.5(−10to12)	4.5(−10to12)	3.3(−9to10.3)	

*IC50, the half maximal inhibitory concentration; ARGP, autophagy-related gene pair.*

### Comparative Analysis of Predictive Values Between ARGP Signature and Published Gene Signatures

**FIGURE 7:**
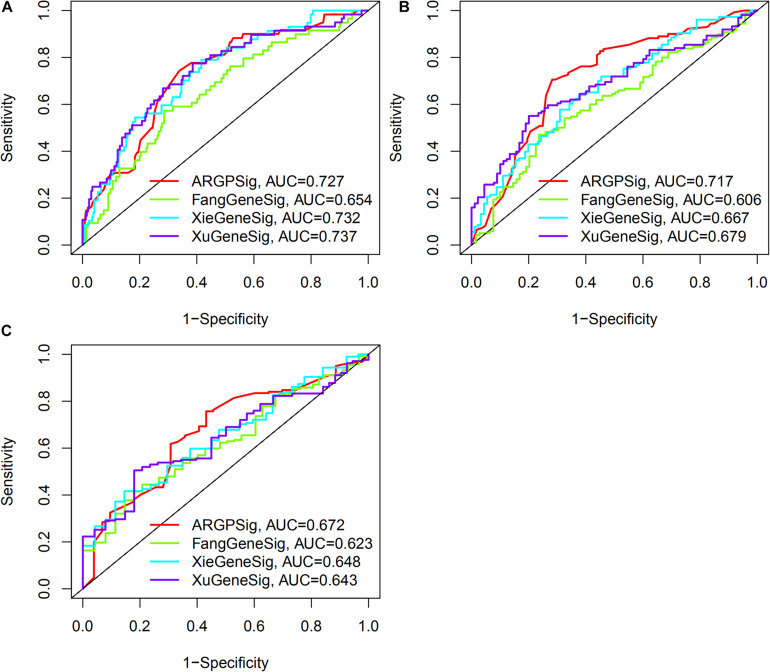
Comparative analysis of the predictive value of ARGP signature with published gene signature. **(A–C)** The AUC at 1-, 3-, 5-years OS of ARGP signature, Fang gene signature, Xie gene signature, and Xu gene signature, respectively.

As shown in Figure 7, the AUC at 1-, 3-, 5-years OS compared between the ARGP signature and 3 published gene signatures in the same TCGA set, which included a 6-gene signature (FangGeneSig), a 7-gene signature (XieGeneSig), and an 8-gene signature (XuGeneSig) (Fang and Chen, 2020; Xie et al., 2020; Xu et al., 2020). Although the AUC of the ARGP signature at 1-year OS was 0.727, which was lower than that of XieGeneSig (0.732) and XuGeneSig (0.737), the AUC of the ARGP signature at 3- and 5-year OS was 0.717 and 0.672, respectively, which was higher than that of FangGeneSig (0.606 and 0.623), XieGeneSig (0.667 and 0.648) and XuGeneSig (0.679 and 0.643). These results suggest that the predictive value of our signature was more accurate than that of the aforementioned 3 published gene signatures in longer OS prediction.

### Establishment of a Nomogram to Predict the OS of HCC Patients

**FIGURE 8:**
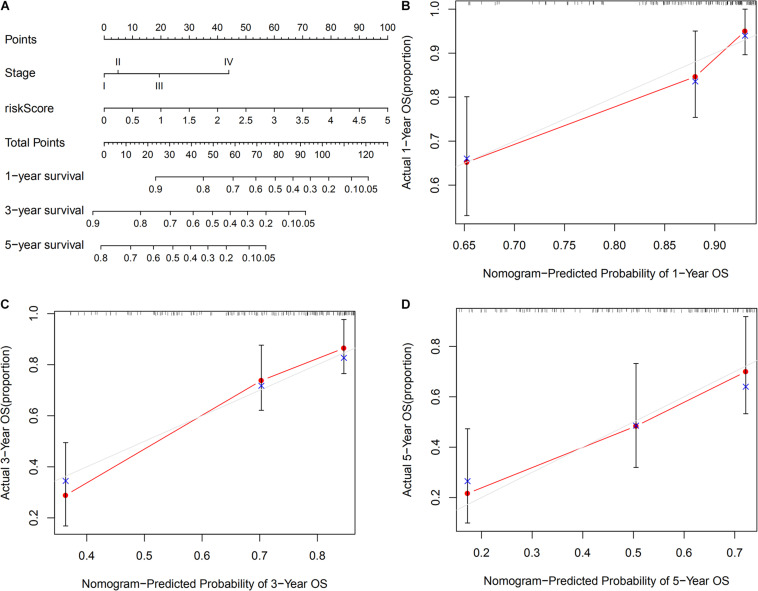
Construction of a nomogram based on risk score and TNM stage in TCGA set. **(A)** A nomogram consists of risk score and stage to predict the OS of HCC patients at 1-, 3-, and 5-years. **(B–D)** Calibration curves to validate the prediction value of nomogram at 1-, 3-, and 5-year OS, respectively.

The TNM stage and risk score of the signature could potentially be independent prognostic factors, as revealed through multivariate Cox regression analysis. Hence, a nomogram that consists of the TNM stage and risk score was constructed, to predict the 1-, 3-, and 5-years OS among patients with HCC. As shown in Figure 8, calibration curves of the nomogram at 1-, 3-, and 5-years OS were proximal to the actual line, indicating that our nomogram performed well in predicting the OS of patients with HCC.

## Discussion

HCC is one of the most prevalent malignancies worldwide. Patients with HCC are suffering high risk for recurrence and metastasis. Over the past decades, *the therapeutic* effect of surgery for HCC and *adjuvant* therapy remained unsatisfactory. With studies on autophagy, attention has been shifting to studies on novel biomarkers for tumor autophagy for estimating treatment responses and survival outcomes.

Hence, based on comprehensive bioinformatics analysis, 6 OS-related ARGPs that could potentially serve as effective biomarkers for HCC were identified. The molecular function of these ARGs was primarily related to the immune function and autophagy, indicating that the potential molecular mechanisms underlying the effect of these ARGs on HCC prognosis are related to immune and autophagy. Furthermore, this 6-ARGP prognostic signature could help doctors to classify patients with HCC into 2 subgroups with significantly different OS. The ROC of the prognostic signature indicated moderate predictive accuracy in OS prediction for patients with HCC, and it revealed an adequate discrimination ability of OS in subgroup analysis, indicating that the prognostic signature was applicable for different subgroups of patients with HCC. Multivariate Cox regression analysis revealed that the risk score of the prognostic signature could serve as an independent prognostic factor. Moreover, a nomogram consisting of the TNM stage and risk score was constructed to better predict the OS of HCC patients more clearly, which performed well in OS prediction.

Although, some ARG based signatures have been published, the methods of our study are different. Most of them only choose ARGs to construct a prognostic signature and validated the signature in other data sets. While we performed pairwise comparison analysis to identify ARGPs and then construct a prognostic signature based on ARGPs. Compared with ARGs, ARGPs can reduce the batch effects when validating the prognostic signature in other data sets, such as ICGC, GEO data set, etc. So, the validated results are more reliable and accurate. In addition, we validate the signature both externally and internally, while the published articles only validate the signature externally. We also perform subgroup analysis to further validate the predictive value of the signature for HCC patients in different clinical features and the results show the signature performs well. To guide the therapy of HCC, a drug sensitivity analysis is performed to identify potential small molecular drugs and 6 drugs are identified. So, the results of our study may be more clinically meaningful compared with published articles, and further researches are needed.

The 6-ARGP signature highlighted 11 ARGs, including BAK1, PELP1, BIRC5, CDKN2A, RGS19, CAPN2, ULK3, DIRAS3, TMEM74, PRKCD, and RB1CC1. Most of these ARGs are correlated with the prognosis of HCC or other cancers. BAK1 is an important cell death regulator that can initiate mitochondria-mediated apoptosis and is reportedly correlated with the occurrence of several cancers (Slager et al., 2012; Wang Y. D. et al., 2013; Marcotte et al., 2017). PELP1 serves as a proto-oncogene in all hormone-responsive cancers, including breast, prostate cancers, ovarian, and other cancers (Dimple et al., 2008; Cortez et al., 2012; Yang et al., 2012; Daniel et al., 2015). BIRC5 overexpression was reported in breast cancer, lung adenocarcinoma, and neuroblastic malignance specimens (Hagenbuchner et al., 2016; Hamy et al., 2016; Cao et al., 2019). In a rat model of HCC, combination therapy with a CDKN2A inhibitor and transarterial chemoembilization promoted cancer cell necrosis (Gade et al., 2017). Wang Y. et al. (2013) reported that RGS19 suppressed the occurrence of non-small-cell carcinoma by downregulating Ras. ULK3 is reportedly involved in cancer-associated fibroblast conversion by activating 2 main signaling pathways (Goruppi et al., 2017). Recent studies have revealed that CAPN2 plays a vital role in tumorigenesis and tumor progression in breast cancer, and colon cancer (Storr et al., 2012; Mo et al., 2019). DIRAS3 is downregulated in 60% of ovarian cancers and negatively related to progression-free survival (Yu et al., 2003; Rosen et al., 2004). Sun Y. et al. (2017) reported that TMEM74 promotes tumor cell survival by inducing autophagy by interacting with ATG16L1 and ATG9A. PRKCD is downregulated in HCC cells and PRKCD upregulation can suppress the viability of HCC cells (Nambotin et al., 2011). RB1CC1 also called FIP200, is crucial in autophagy and is associated with the prognosis of and drug resistance in multiple cancers, including HCC (Yeo et al., 2020). Our results show that the aforementioned ARGs are correlated with the prognosis of HCC; However, the underlying molecular mechanism of these ARGs in HCC prognosis requires further investigation.

To explore the mechanisms through which the ARGP signature effectively stratifies patients with HCC, the expression profiles of CTLA4 and LC3 between the high- and low-risk groups was performed. CTLA4 is a receptor on the surface of activated T cells and act as an effective immune therapy checkpoint, whose functions are to inhibit the production of IL-2, proliferation of T cells, and cell cycle (Fraser et al., 1999; Intlekofer and Thompson, 2013; Bhandaru and Rotte, 2019). LC3 is essential for the execution of autophagy. Therefore it is a widely accepted marker for autophagy, which can be a potential target for anticancer therapy (Schaaf et al., 2016). These results show that the low-risk group has a lower expression level of CTLA4 and a higher expression level of LC3. CTLA4 and LC3 dysregulation may be responsible for the difference in survival outcomes between the high- and low-risk groups.

Based on our results, we hypothesize that immunological and autophagy-related small-molecule drugs might be used to treat patients with HCC. Hence, a drug sensitivity analysis was performed to explore potentially effective small-molecule drugs for patients with HCC. 6 drugs were identified, including axitinib, rapamycin, temsirolimus, docetaxel, metformin, and bleomycin. Recent studies have reported that axitinib serves as a multi-receptor tyrosine kinase inhibitor to treat multiple cancers, and axitinib inhibits the VEGF receptor, platelet-derived growth factor receptor, and epidermal growth factor receptor (Ongkeko et al., 2005; Bran et al., 2009; Lawrence et al., 2015). Lin et al. (2020) reported that axitinib is an effective second-line therapy drug for advanced patients with HCC, who failed sorafenib therapy. Rapamycin and temsirolimus belong to Rapalogs, have been reported to suppress proliferation and promote autophagy in HCC cells by targeting the mTOR signaling pathway (Lu et al., 2020). In the work of Hui et al. (2010), rapamycin and temsirolimus can significantly inhibit the growth and metastasis of PLC/PRF/5 human HCC cells. Docetaxel belongs to the taxane family, and preclinical studies have reported the anticancer potential of docetaxel in suppressing HCC cell proliferation. For example, docetaxel treatment can reduce the tumor size in a nude mouse model of HCC, and suppress the proliferation capacity of the HepG2 cell line (Zhu et al., 2016). In addition, Zhang et al. (2019) revealed that docetaxel can induce HCC cell apoptosis by inhibiting the PI3K/AKT signaling pathway. Metformin is used to treat not only diabetes but also tumors. For instance, metformin can inhibit tumor cell proliferation by targeting mTOR complex 1 via an AMPK-independent mechanism (Kalender et al., 2010). DeWaal et al. (2018) reported that metformin can induce cell cycle arrest in HCC cells by targeting mTOR complex 1 through an AMPK-independent mechanism as well. These studies indicate that the AMPK-independent anticancer activities of metformin may be a novel finding Overall, this study and other preclinical studies have revealed that these small- molecule drugs can be potentially effective drugs in treating HCC, and further clinical trials are needed to validate these results.

In this study, we identified an ARGP based prognostic signature that performs well in predicting the OS of patients with HCC. For all we know, this is the first reported ARGP-based signature for HCC. However, our study has several limitations. First, the results were biased to an extent because we used fewer non-tumor specimens than HCC specimens. Second, the underlying molecular mechanisms of HCC in this study have not been determined on the basis of *in vitro* and *in vivo* studies. Further studies are needed to validate these results.

## Conclusion

An ARGP prognostic signature was identified and validated in different data sets, this signature performed better in OS prediction of HCC in comparison with 3 previously published gene signatures. Furthermore, 6 small-molecule drugs were identified to be potentially effective drugs in treating HCC.

## Data Availability Statement

Publicly available datasets were analyzed in this study. This data can be found here: The data that support the findings of this study are openly available in TCGA data portal (https://portal.gdc.cancer.gov/), ICGC data base (https://dcc.icgc.org/), and HADb (http://www.autophagy.lu/).

## Author Contributions

SQL: conceptualization and manuscript revision. GPZ, ZBS, and YY: data curation. ZBS: data analysis and figure plot. ZBS and GPZ: manuscript writing. All authors final approval of manuscript.

## Conflict of Interest

The authors declare that the research was conducted in the absence of any commercial or financial relationships that could be construed as a potential conflict of interest.

## Publisher’s Note

All claims expressed in this article are solely those of the authors and do not necessarily represent those of their affiliated organizations, or those of the publisher, the editors and the reviewers. Any product that may be evaluated in this article, or claim that may be made by its manufacturer, is not guaranteed or endorsed by the publisher.

## Footnotes

1
https://cancergenome.nih.gov/

2
https://dcc.icgc.org/

3
http://www.autophagy.lu/

4
https://www.cancerrxgene.org

5
https://github.com/paulgeeleher/pRRophetic

6
https://cran.r-project.org/

7
https://strawberryperl.com/

## Supplementary Material

Supplementary Figure 1 Risk score analysis of the prognostic signature in different cohorts. **(A)** Heatmap plot represents the expression of the 6 ARGPs between high- and low-risk groups in the training set, test set, TCGA set, and ICGC set, respectively. **(B)** Survival status of patients in the train set, test set, TCGA set, and ICGC set, respectively. **(C)** The rank of the risk score in the training set, test set, TCGA set, and ICGC set, respectively.

Supplementary Figure 2 Subgroup analysis in the training set and ICGC set. **(A,B)** The KM curve represents the OS of high- and low-risk groups in different subgroups of the training set. **(C)** The KM curve represents the OS of high- and low-risk groups in different subgroups of the ICGC set.

Supplementary Figure 3 Subgroup analysis in the test set and TCGA set. **(A,B)** The KM curve represents the OS of high- and low-risk groups in different subgroups of the test set. **(C,D)** The KM curve represents the OS of high- and low-risk groups in different subgroups of the TCGA set.

Supplementary Figure 4 Clinical relevance analysis of risk score. **(A–D)** The relationship of stage group with risk score in the training set, test set, TCGA set, and ICGC set, respectively. **(E–G)** The relationship of grade group with risk score in the training set, test set, and TCGA set, respectively.
