# Supplementary figures and images for: A Prognostic Autophagy-Related Gene Pair Signature and Small-Molecule Drugs for Hepatocellular Carcinoma
Source: Front Genet. 2021 Aug 23;12:689801. doi: 10.3389/fgene.2021.689801 (PMC8419440; doi:10.3389/fgene.2021.689801)

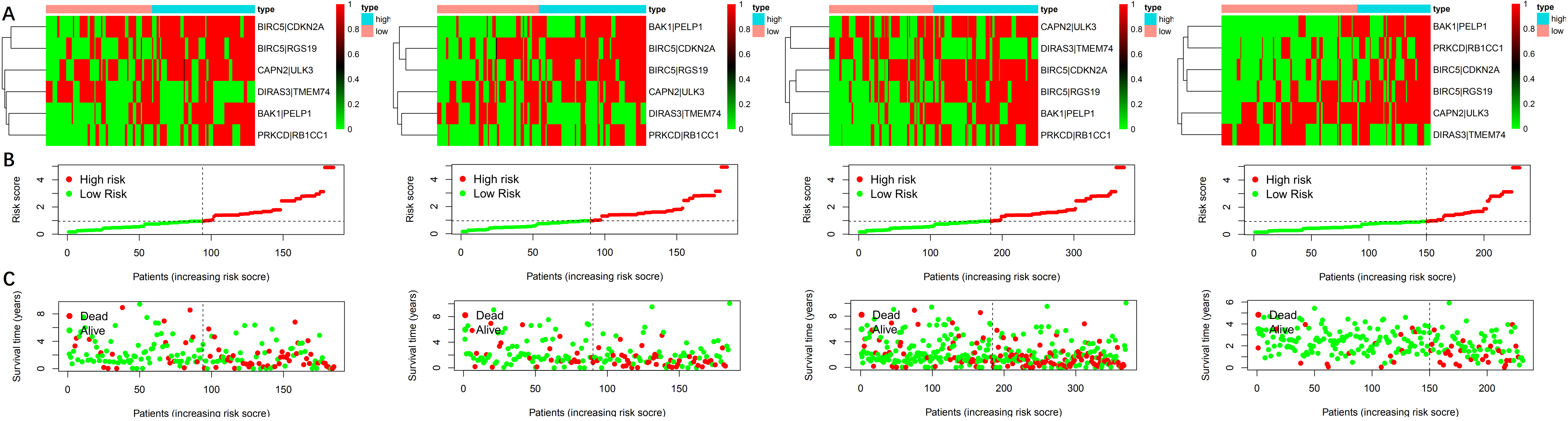

Supplement: Supplementary Figure 1 — Risk score analysis of the prognostic signature in different cohorts. (A) Heatmap plot represents the expression of the 6 ARGPs between high- and low-risk groups in the training set, test set, TCGA set, and ICGC set, respectively. (B) Survival status of patients in the train set, test set, TCGA set, and ICGC set, respectively. (C) The rank of the risk score in the training set, test set, TCGA set, and ICGC set, respectively. [file Image_1.TIF]

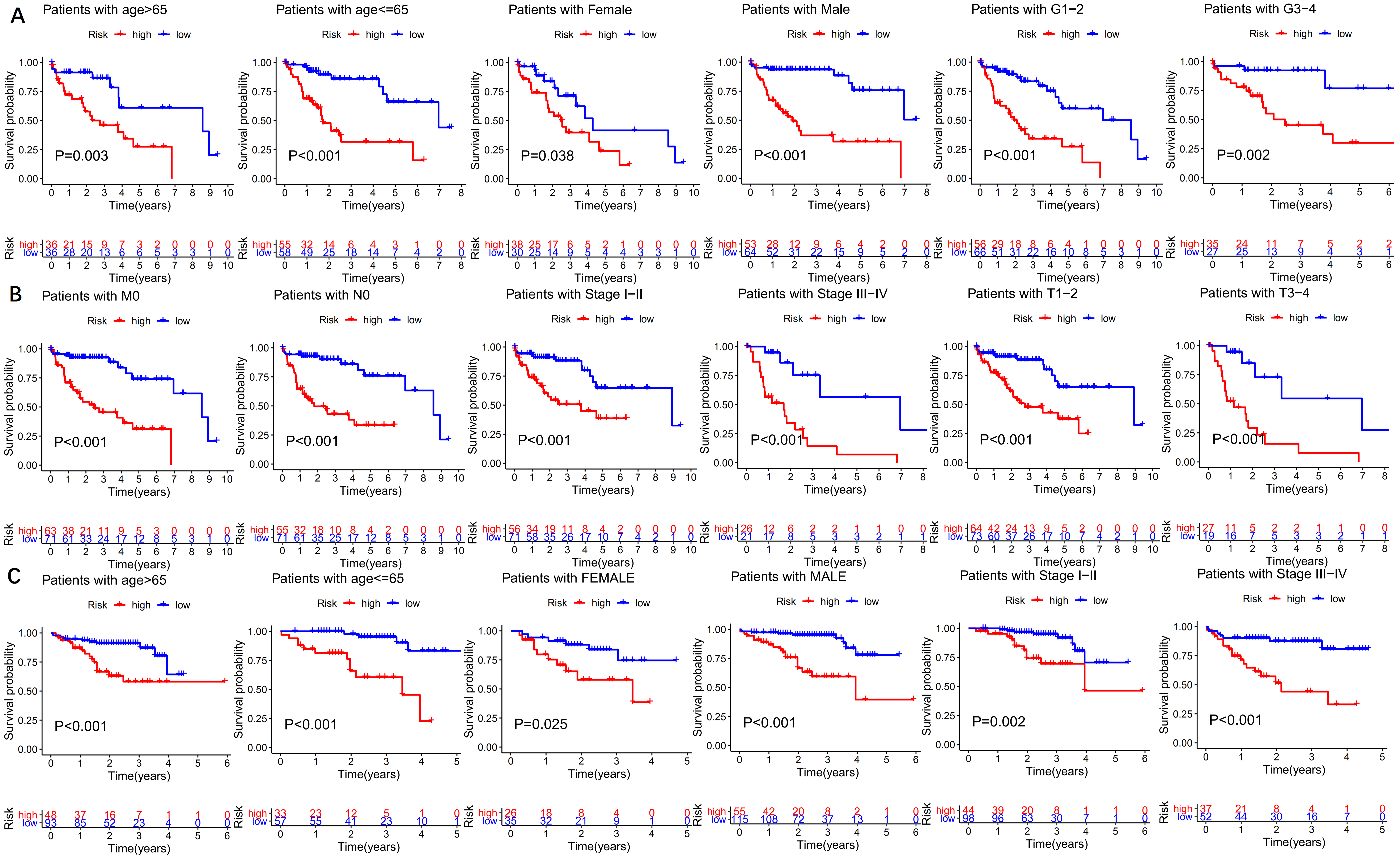

Supplement: Supplementary Figure 2 — Subgroup analysis in the training set and ICGC set. (A,B) The KM curve represents the OS of high- and low-risk groups in different subgroups of the training set. (C) The KM curve represents the OS of high- and low-risk groups in different subgroups of the ICGC set. [file Image_2.TIF]

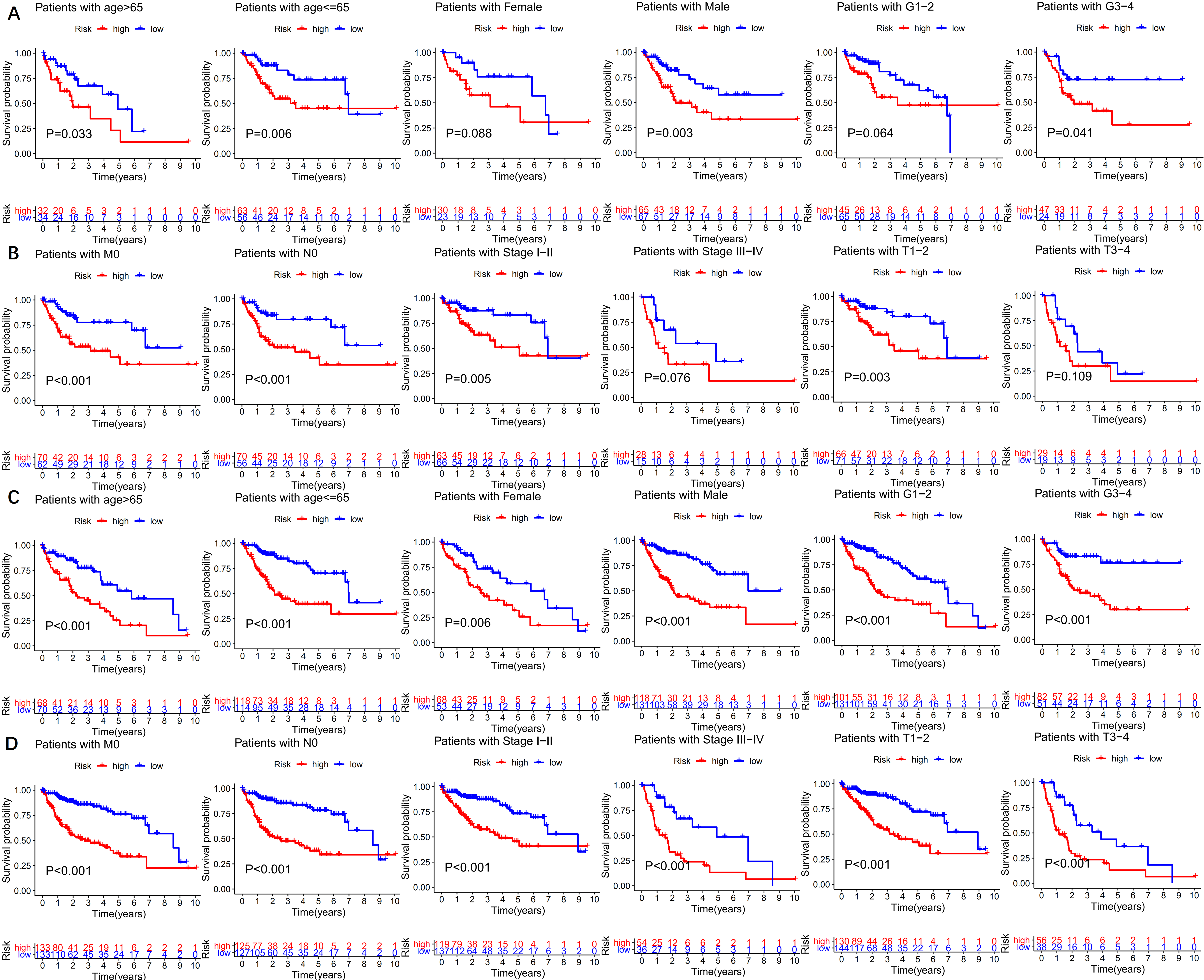

Supplement: Supplementary Figure 3 — Subgroup analysis in the test set and TCGA set. (A,B) The KM curve represents the OS of high- and low-risk groups in different subgroups of the test set. (C,D) The KM curve represents the OS of high- and low-risk groups in different subgroups of the TCGA set. [file Image_3.TIF]

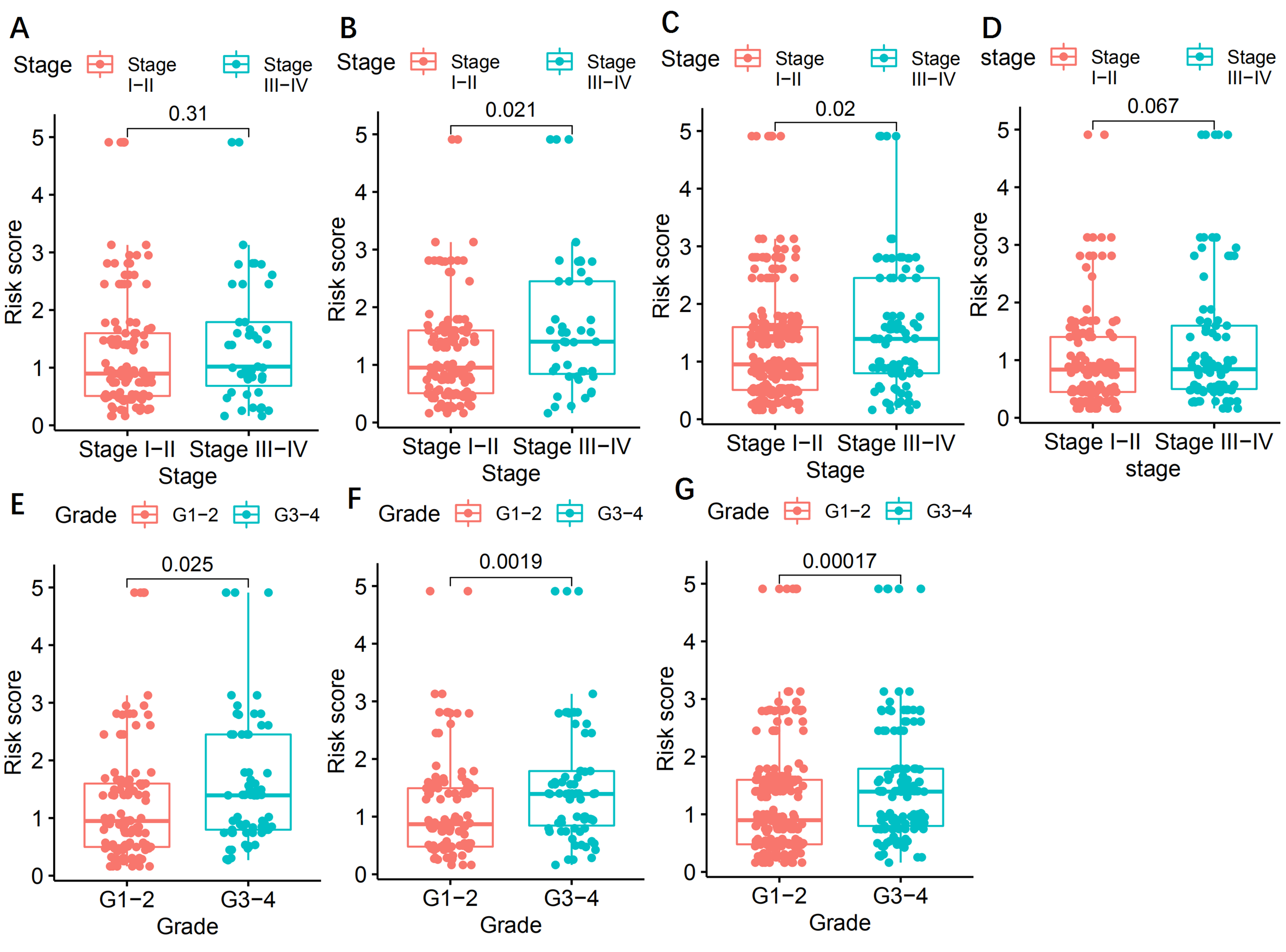

Supplement: Supplementary Figure 4 — Clinical relevance analysis of risk score. (A–D) The relationship of stage group with risk score in the training set, test set, TCGA set, and ICGC set, respectively. (E–G) The relationship of grade group with risk score in the training set, test set, and TCGA set, respectively. [file Image_4.TIF]
